# Involvement of the Kynurenine Pathway in Human Glioma Pathophysiology

**DOI:** 10.1371/journal.pone.0112945

**Published:** 2014-11-21

**Authors:** Seray Adams, Charles Teo, Kerrie L. McDonald, Anna Zinger, Sonia Bustamante, Chai K. Lim, Gayathri Sundaram, Nady Braidy, Bruce J. Brew, Gilles J. Guillemin

**Affiliations:** 1 MND and Neurodegenerative Diseases Research Centre, Australian School of Advanced Medicine, Macquarie University, Sydney, NSW, Australia; 2 Centre for Minimally Invasive Neurosurgery, Prince of Wales Hospital, Sydney, NSW, Australia; 3 Cure For Life Neuro-Oncology Group, Lowy Cancer Research Centre, University of New South Wales, Sydney, NSW, Australia; 4 School of Medical Sciences, University of Sydney, Sydney, NSW, Australia; 5 Bioanalytical Mass Spectrometry Facility, University of New South Wales, Sydney, NSW, Australia; 6 Centre for Healthy Brain Ageing, School of Psychiatry, University of New South Wales, Sydney, NSW, Australia; 7 St Vincent's Centre for Applied Medical Research, Sydney, NSW, Australia; 8 Department of Neurology, St Vincent's Hospital, Sydney, NSW, Australia; 9 Department of Pharmacology, School of Medical Sciences, University of New South Wales, Sydney, NSW, Australia; Imperial College London, United Kingdom

## Abstract

The kynurenine pathway (KP) is the principal route of L-tryptophan (TRP) catabolism leading to the production of kynurenine (KYN), the neuroprotectants, kynurenic acid (KYNA) and picolinic acid (PIC), the excitotoxin, quinolinic acid (QUIN) and the essential pyridine nucleotide, nicotinamide adenine dinucleotide (NAD^+^). The enzymes indoleamine 2,3-dioxygenase-1 (IDO-1), indoleamine 2,3-dioxygenase-2 (IDO-2) and tryptophan 2,3-dioxygenase (TDO-2) initiate the first step of the KP. IDO-1 and TDO-2 induction in tumors are crucial mechanisms implicated to play pivotal roles in suppressing anti-tumor immunity. Here, we report the first comprehensive characterisation of the KP in 1) cultured human glioma cells and 2) plasma from patients with glioblastoma (GBM). Our data revealed that interferon-gamma (IFN-γ) stimulation significantly potentiated the expression of the KP enzymes, IDO-1 IDO-2, kynureninase (KYNU), kynurenine hydroxylase (KMO) and significantly down-regulated 2-amino-3-carboxymuconate semialdehyde decarboxylase (ACMSD) and kynurenine aminotransferase-I (KAT-I) expression in cultured human glioma cells. This significantly increased KP activity but significantly lowered the KYNA/KYN neuroprotective ratio in human cultured glioma cells. KP activation (KYN/TRP) was significantly higher, whereas the concentrations of the neuroreactive KP metabolites TRP, KYNA, QUIN and PIC and the KYNA/KYN ratio were significantly lower in GBM patient plasma (n = 18) compared to controls. These results provide further evidence for the involvement of the KP in glioma pathophysiology and highlight a potential role of KP products as novel and highly attractive therapeutic targets to evaluate for the treatment of brain tumors, aimed at restoring anti-tumor immunity and reducing the capacity for malignant cells to produce NAD^+^, which is necessary for energy production and DNA repair.

## Introduction

Gliomas are the most common primary central nervous system (CNS) tumor of the brain [1]. In the current World Health Organization (WHO) 2007 version, diffuse astrocytomas are classified according to a three-tiered grading system (grades II, III and IV); diffuse astrocytoma (grade II; AII), anaplastic astrocytoma (grade III; AIII), and glioblastoma (grade IV; GBM). Oligodendrogliomas are divided into 2 grades: diffuse oligodendroglioma (grade II; OII) and anaplastic oligodendroglioma (grade III; OIII) [1]. GBM is by far, the most prevalent and most malignant type of primary brain tumor in adults [2]. GBM is an aggressive tumor that progresses rapidly, and patients with GBM have a dismal prognosis; the median survival of GBM patients is approximately 14.6 months with maximal treatment [3], but those without any intervention die soon after diagnosis [4]. This highlights the importance of developing novel pharmacological therapies with greater clinical efficacy than those that are currently available.

**Figure 1:**
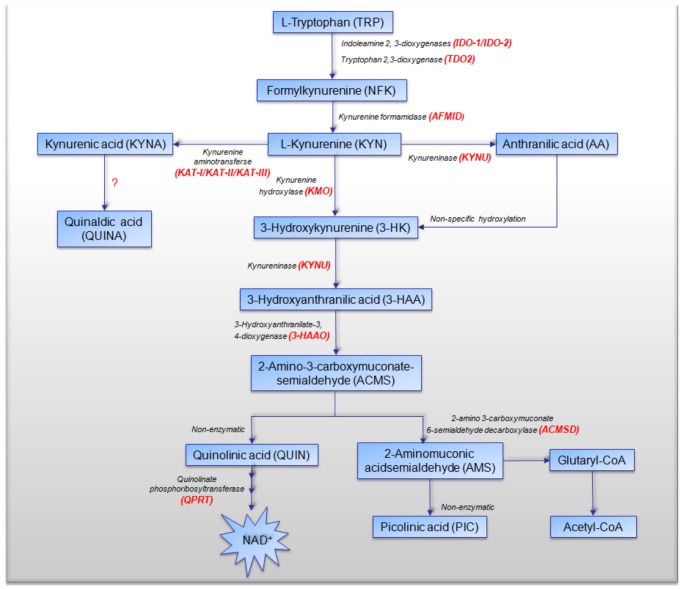
Schematic diagram of tryptophan catabolism along the KP (adapted from [60]). The kynurenine pathway (KP) is a major degradative pathway of TRP that ultimately leads to production of NAD^+^.

The catabolism of L-tryptophan (TRP) is accurately controlled by a number of metabolic pathways. As the major route of TRP catabolism, KP metabolism leads to the production of the essential pyridine nucleotide, nicotinamide adenine dinucleotide (NAD^+^), and a number of neuro-active metabolites, including kynurenine (KYN), the neurotoxic free radical generator 3-hydroxykynurenine (3-HK), 3-hydroxyanthranilic acid (3-HAA), the excitatory N-methyl- D-aspartate (NMDA) receptor agonist and neurotoxin, quinolinic acid (QUIN), and the neuroprotectants, kynurenic acid (KYNA) and picolinic acid (PIC) (Figure 1). KYN is hydroxylated by kynurenine hydroxylase (KMO), resulting in 3-HK, which then undergoes hydrolytic cleavage of the Cβ–Cγ bond, catalysed by kynureninase (KYNU), to give 3-HAA [5]. The mechanisms of QUIN-induced neuronal toxicity are multifactorial. These have been well reviewed elsewhere (reviewed by [6]). In contrast to the neurotoxic activity of QUIN, KYNA and PIC exhibit neuroprotective effects [7]. In addition, KYNA behaves as a non-competitive antagonist of the α7 nicotinic acetylcholine receptor [8] and can directly activate the G-protein coupled receptor GPR35 *in vitro* [9]. KYNA has also recently been found to be a potent reactive oxygen species scavenger in a mechanism likely independent of its antagonistic activity on NMDA and nicotinic receptors [10].

In mammals, three different heme-enzymes catalyse the first, rate-limiting step in the catabolism of tryptophan via the KP to produce NFK: tryptophan 2,3-dioxygenase-2 (TDO-2), indoleamine 2,3-dioxygenase (IDO-1), and the IDO-1-related enzyme, IDO-2 (or INDOL1) [11]. The release of the pro-inflammatory cytokine, interferon-γ (IFN-γ), represents the most potent inducer of IDO-1, switching on gene expression and activity [12]. IDO-1 has been the focus of attention in recent years because of its immunosuppressive effects on T lymphocytes, resulting partly from tryptophan depletion and partly from direct effects of tryptophan catabolites [13], [14]. IDO-1 expression has been suggested to play a pivotal role in the inhibition of tumor-specific immunity [15]–[17]. Studies also suggest that IDO-1 expression correlates with and may be a significant predictor of poor clinical prognosis in patients with various cancers [16], [18], [19]. IDO-1 expression has been shown in nine of ten human GBM biopsies [15] and in brain tumor cell lines [20] and GBM cell lines [21]. More recently, TDO-2 has been implicated in mediating immunosuppressive effects similar to those of IDO-1 [14], [22]. TDO-2-dependent production of KYN by gliomas might be a novel mechanism for suppressing anti-tumor immunity and supporting tumor growth through activation of the AhR [23]. A reciprocal relationship was recently found in an orthotopic GL261 cell-based tumor model, whereby IDO-competent brain tumors were infiltrated by an increased frequency of the immunosuppressive CD4^+^FoxP3^+^GITR^+^ regulatory T cells (Tregs), coincident with a decreased frequency of CD8^+^ cytotoxic T cells when compared with IDO-deficient brain tumors [24]. 3-HAA has the ability to cause a reduction in CD4^+^Th17 cells and a reciprocal increase in the fraction of Tregs [25]. The loss of Th17/Treg balance was suggested to be mediated directly by 3-HAA from IDO-1 induction by myeloid antigen-presenting dendritic cells (DCs) (Favre et al., 2010). Interestingly, induction of a tolerogenic phenotype in naive CD4^+^ T cells result from the combined effects of tryptophan starvation and tryptophan metabolites [26]. Upregulation of IDO expression by brain tumors was also associated with a significantly worse prognosis in patients [24]. However, to date, the involvement of the downstream machinery of the KP in brain tumor pathophysiology has been virtually unexplored.

Currently, a comprehensive evaluation of the KP metabolic profile, and a full characterisation of KP enzyme expression in human glioma has not, to our knowledge, been previously explored. Herein, we present the first comprehensive characterisation of the KP in an *in vitro* model using a series of cultured human glioma samples. We determined the mRNA expression of 12 KP enzymes and analysed the production of KP metabolites in normal versus inflammatory conditions (addition of IFN-γ) in cultured glioma cells in comparison with the normal ‘healthy’ human foetal astrocytes (HFA) and human adult astrocytes (AA). We also present the first comprehensive evaluation of the KP metabolic profile in a series of 18 GBM patient plasma samples versus plasma samples from 18 healthy subjects as controls. These findings provide renewed evidence implicating the involvement of the KP in glioma pathophysiology, and further increase our understanding into the relationship between the KP and human glioma. Inhibiting KYNU and KMO may represent a novel therapeutic approach for the treatment of glioma.

## Results

### Cell Cultures

**Figure 2:**
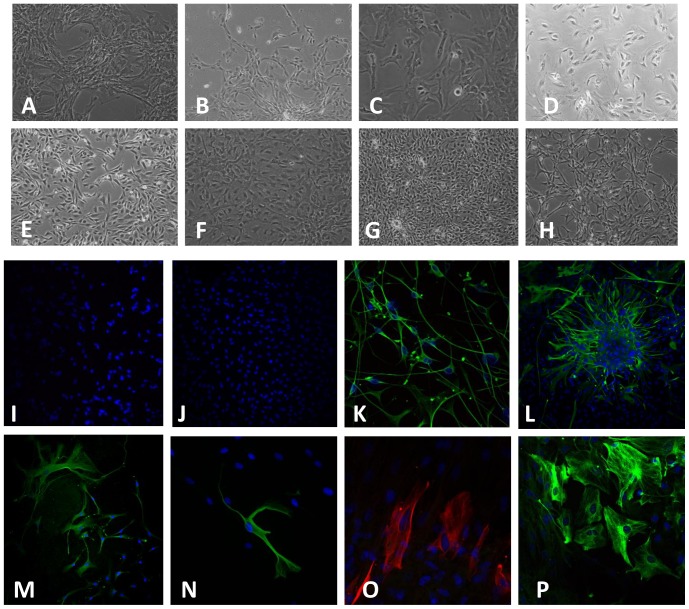
Phase contrast microscopy images of HFA and brain tumor cells and immunofluorescence images of cultured primary brain tumor cells, AA and HFA. HFA were derived from three different foetal brains. (**A**) 18-week foetus (10× magnification); (**B**) 19-week foetus, first week in culture (10× magnification); (**C**) 19-week foetus, day 5 in culture (20× magnification); (**D**) Primary GBM after 2 trypsinisations (20x); (**E**) Recurrent GBM (20x); (**F**) Secondary GBM after 3 trypsinisations (10x); (G) OII 14 days in culture (10x); (**H**) AII 13 days in culture (10x); (**I**) Negative control for NGS merged with DAPI; (**J**) Negative control for secondary antibodies merged with DAPI; (**K**) GBM cells derived from one patient – GFAP (DAKO) merged with DAPI and CD68 (zoomed: 2.4× magnification); (**L**) primary brain tumor cells from one patient (male, 40 years old) (passaged three times) - GFAP (Novocastra) merged with DAPI; (**K and L**) GFAP (green) was used as a GBM marker and CD68 (red) was used as a marker for microglia. (**M**) AA from one Female, 61 years old; (passaged twice) - GFAP (Novocastra) merged with DAPI; (**N**) AA from one male 40 years old; (passaged once) - GFAP (Sigma) merged with DAPI and CD68 (Abcam); (**M and N**) GFAP (green) was used as an astrocyte marker and CD68 (red) and CD11b (green) were used as markers for microglia; (**O**) HFA from one 17-week- old foetus (passaged once) - GFAP (Novocastra) merged with DAPI and CD11b (Novus); (**P**) HFA from an 18-week- old foetus (passaged once) - GFAP (DAKO) merged with DAPI and CD68 (zoomed: 2.1× magnification); (**O and P**) GFAP (green and red) was used as an astrocyte marker and CD68 (red) and CD11b (green) were used as markers for microglia. Nuclei indicated by DAPI (blue) in all images.

Cells derived from biopsied tissue from human foetal and adult brain and glioma tissue were grown as monolayer cultures (Figure 2 (A–H)). Cultures of purified HFA, AA and glioma cells were immunostained with the immunocytochemical marker glial fibrillary acidic protein (GFAP) to verify the cells were of astrocytic lineage (Figure 2 (I–P)). The presence of microglia in cultures was determined by immunocytochemical labelling with the microglial markers CD68 and CD11b. Analysis revealed strong immunostaining of the GFAP marker in HFA and glioma cell cultures, while AA cultures showed very weak GFAP expression in the majority of cultures (data not shown). These cells may suggest the presence of GFAP-negative astrocytes [27]. Although we were unable to determine the exact proportions of astrocytes, glioma cells and microglia in this study, ICC showed no immunofluorescence for the microglial markers, CD68 and CD11b, in the majority of cultures. Contamination of microglia was excluded in all other cultures by showing negative staining for CD68 and CD11b. It is important to note that the presence of microglia was not determined in all cultures grown. Immunostaining with DAPI showed only the presence of the nucleus in all cultures.

### Quantitative qRT-PCR to detect relative mRNA transcript expression of KP enzymes

**Figure 3:**
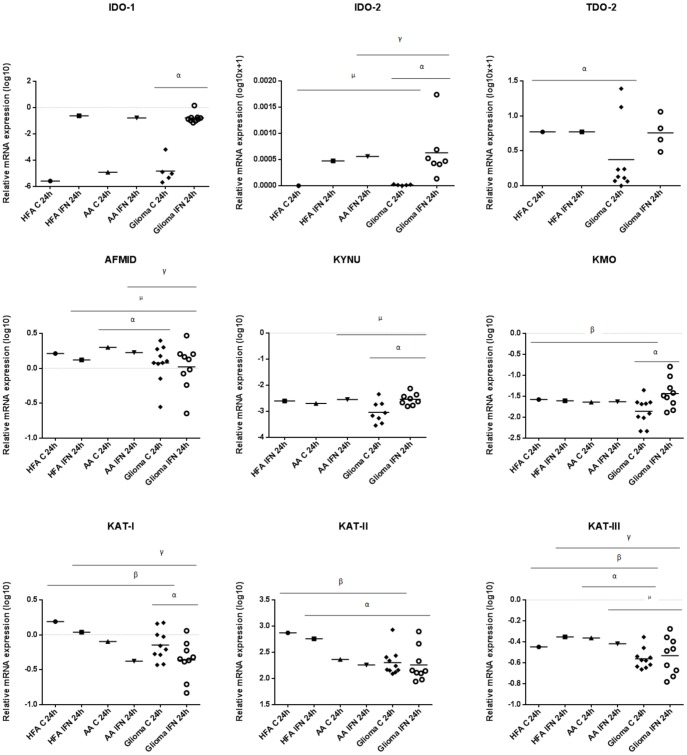
Relative mRNA expression of KP enzymes in HFA and AA versus glioma using qRT-PCR. The graphs indicate the relative mRNA expression normalised to HPRT mRNA expression. All cell cultures were untreated (C) and treated with IFN-γ (IFN) for 24 hours. Graphs indicate Log10 and Log10 (x+1) of the quantified values. IDO-1 expression in stimulated glioma cells (n = 9) compared to unstimulated (n = 5) (^α^p<0.0001). IDO-2 expression in stimulated glioma cells (n = 7) compared to unstimulated (n = 5) (^α^p<0.05); IDO-2 expression in unstimulated glioma cells compared to unstimulated HFA (^µ^p<0.05); IDO-2 expression in stimulated glioma cells compared to stimulated AA (^γ^p<0.05). TDO-2 expression in unstimulated glioma cells (n = 9) compared to unstimulated HFA (^α^p<0.05). AFMID expression in unstimulated glioma cells (n = 10) compared to unstimulated AA (^α^p<0.05); AFMID expression in stimulated glioma cells (n = 9) compared to stimulated AA cells (^γ^p<0.05); AFMID expression in stimulated glioma cells compared to stimulated HFA (^µ^p<0.05). KYNU expression in stimulated glioma compared to unstimulated (n = 8) (^α^p<0.01); KYNU expression in stimulated glioma cells (n = 8) compared to stimulated AA cells (^µ^p<0.01). KMO expression in stimulated glioma (n = 9) compared to unstimulated (n = 10) (^α^p = 0.007); KMO expression in unstimulated glioma cells compared to unstimulated HFA cells (^β^p<0.05). KAT-I expression in stimulated glioma cells (n = 9) compared to unstimulated (n = 10) (^α^p<0.05); KAT-I expression in unstimulated and stimulated glioma cells compared to unstimulated and stimulated HFA cells, respectively (^βγ^p<0.01). KAT-II expression in unstimulated glioma cells (n = 10) compared to unstimulated HFA (^β^p<0.0001); KAT-II expression in stimulated glioma cells (n = 9) compared to stimulated HFA cells (^α^p = 0.001). KAT-III expression in unstimulated glioma cells (n = 10) compared to unstimulated HFA (^β^p = 0.0042); KAT-III expression in unstimulated glioma cells compared to unstimulated AA cells (^α^p<0.0001); KAT-III expression in stimulated glioma cells (n = 9) compared to stimulated HFA cells (^γ^p = 0.0013); KAT-III expression in stimulated glioma cells compared to stimulated AA cells (^µ^p<0.05).

**Figure 4:**
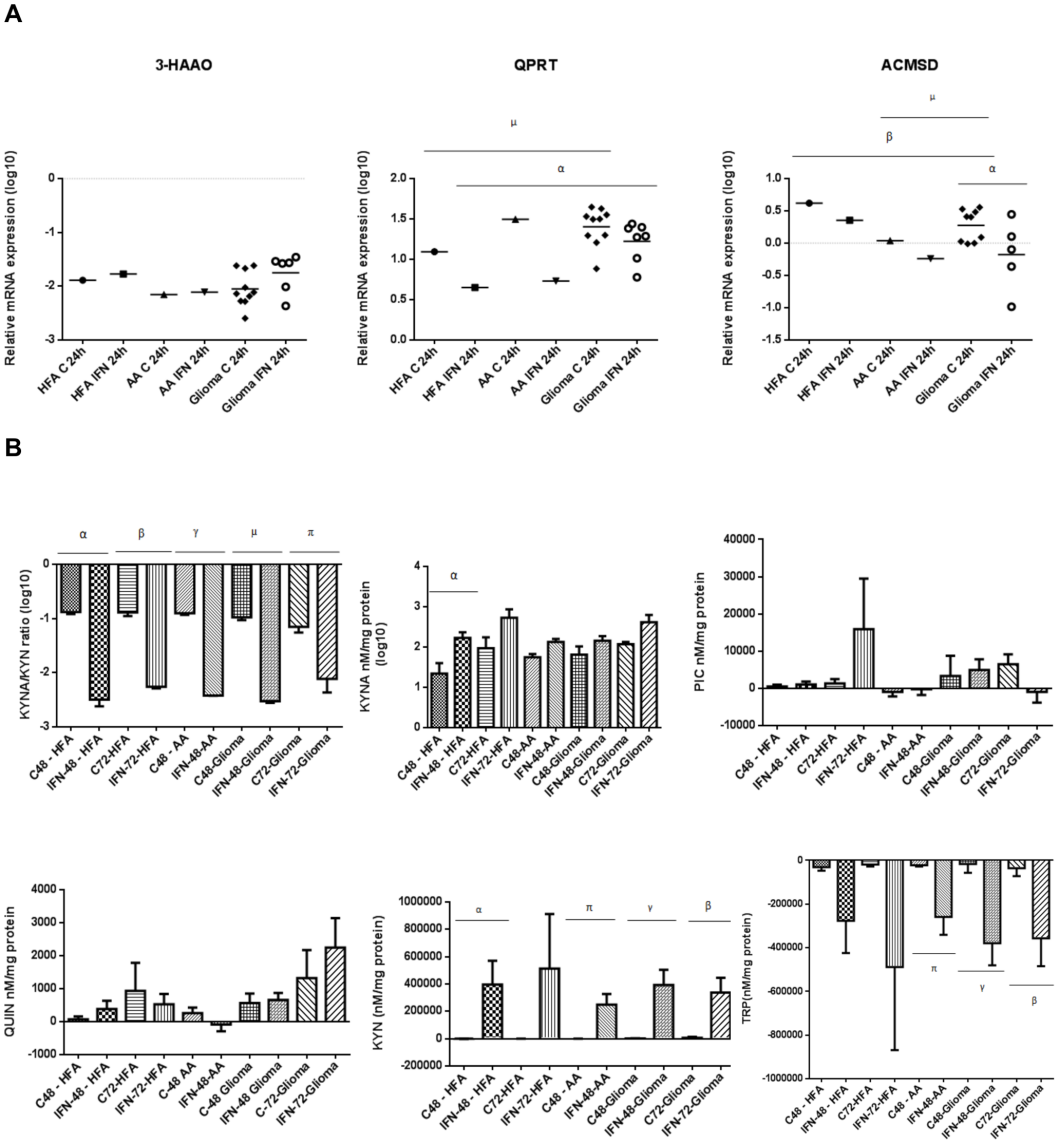
Relative mRNA expression of KP enzymes in HFA and AA versus glioma using qRT-PCR and analysis of the KYNA/KYN ratio and KYNA, PIC, QUIN, KYN and TRP concentrations in the cell culture supernatants of glioma, HFA and AA cells. (**A**) The dot plots indicate the relative mRNA expression normalised to HPRT mRNA expression; All cell cultures were untreated (C) and treated with IFN-γ (IFN) for 24 hours; Graphs indicate Log10 of the quantified values. No statistical significance was observed for 3-HAAO expression; QPRT expression in unstimulated glioma (n = 10) compared to unstimulated HFA cells (^µ^p = 0.0024); QPRT expression in stimulated glioma cells (n = 7) compared to stimulated HFA cells (^α^p = 0.0015). ACMSD expression in stimulated glioma cells (n = 5) compared to unstimulated (^α^p<0.05); ACMSD expression in unstimulated glioma cells (n = 9) compared to unstimulated HFA cells (^β^p = 0.0029); ACMSD expression in unstimulated glioma cells compared to unstimulated AA (^µ^p<0.05). (**B**) Glioma cell cultures for the KYNA/KYN ratio and KYNA concentrations were untreated (n = 7) and treated with IFN-γ (n = 6) for 48 hours and untreated (n = 6) and treated with IFN-γ for 72 hours (n = 6); HFA cell cultures for the analysis of the KYNA/KYN ratio and KYNA concentrations were untreated (n = 8) and treated with IFN-γ (n = 8) for 48 hours and untreated (n = 5) and treated with IFN-γ for 72 hours (n = 5); All AA cell cultures were untreated (n = 3) and treated with IFN-γ (n = 3) for 48 hours; Bar graphs for the KYNA/KYN ratio and KYNA concentrations indicate Log10 of the quantified values; The KYNA/KYN ratio in glioma, HFA and AA cell cultures after 48 and 72 hours of IFN-γ stimulation compared to untreated cultures (^αβγµπ^p<0.001); KYNA concentrations in HFA cell cultures when treated with IFN-γ for 48 hours compared to when untreated after 48 hours (^α^p<0.05); Glioma cell cultures for the analysis of QUIN and PIC concentrations were untreated (n = 7) and treated with IFN-γ (n = 6) for 48 hours and untreated (n = 5) and treated with IFN-γ for 72 hours (n = 5); HFA cell cultures for the analysis of QUIN and PIC concentrations were untreated (n = 10) and treated with IFN-γ (n = 9) for 48 hours and untreated (n = 5) and treated with IFN-γ for 72 hours (n = 5); No statistical significance was observed for QUIN and PIC; Glioma cell cultures for the analysis of KYN and TRP concentrations were untreated (n = 7) and treated with IFN-γ (n = 6) for 48 hours and untreated (n = 6) and treated with IFN-γ for 72 hours (n = 5); HFA cell cultures for the analysis of KYN and TRP concentrations were untreated (n = 9) and treated with IFN-γ (n = 9) for 48 hours and untreated (n = 5) and treated with IFN-γ for 72 hours (n = 5); KYN production in glioma cell cultures after 48 and 72 hours of IFN-γ stimulation compared to untreated cultures after 48 and 72 hours (^γβ^p<0.01); KYN production in HFA cell cultures after 48 hours of IFN-γ stimulation compared to untreated cultures after 48 hours (^α^p<0.05); KYN production in AA after 48 hours of IFN-γ stimulation compared to the untreated cultures (^π^p<0.05); TRP catabolism in AA cell culture supernatant after 48 hours of IFN-γ stimulation compared to untreated cultures after 48 hours (^π^p<0.05); TRP catabolism in glioma cell culture supernatant after 48 and 72 hours of IFN-γ stimulation compared to untreated cultures after 48 hours (^γβ^p<0.05). No other statistical significance was observed.

The expression of KP enzyme expression was measured in a series of cultured HFA, AA and cultured primary glioma patient samples using quantitative qRT-PCR analysis (Figure 3 and Figure 4A). These results are summarised in Table 1.

**Table 1:** Summary of relative mRNA expression of KP enzymes in HFA and AA versus glioma using qRT-PCR.

KP relative expression	HFA C 24 h	HFA IFN 24 h	AA C 24 h	AA IFN 24 h	Glioma C 24 h	Glioma IFN 24 h
**IDO-1**	2.619E-06	0.239	1.205E-05	0.165	0.00013±0.00013	0.27515±0.14413 **↑compared to Glioma C 24 h**
**IDO-2**	No expression	0.001	-	0.001	3.267E-05±1.08698E-05 **↑compared to HFA C**	0.001±0.000 **↑compared to Glioma C and AA IFN**
**TDO-2**	4.892	4.920	-	-	4.246±2.745 **↓compared to HFA C**	5.427±1.829
**AFMID**	1.629	1.319	1.999	1.682	1.368±0.202 **↓compared to AA C**	1.283±0.261 **↓compared to AA IFN and HFA IFN**
**KYNU**	-	0.003	0.002	0.003	0.001 ±0.001	0.003±0.001 **↑compared to Glioma C ↓compared to AA IFN**
**KMO**	0.026	0.025	0.023	0.023	−0.017±0.004 **↓compared to HFA C**	0.051±0.016 **↑compared to Glioma C**
**KAT-I**	1.548	1.089	0.803	0.420	0.799±0.130 **↓compared to HFA C**	0.512±0.100 **↓compared to Glioma C and HFA IFN**
**KAT -II**	742.217	575.129	231.443	180.749	245.606±69.010 **↓compared to HFA C**	242.803±78.131 **↓compared HFA IFN**
**KAT - III**	0.356	0.443	0.433	0.381	0.280±0.022 **↓compared to HFA C and AA C**	0.316±0.042 **↓compared to HFA IFN and AA IFN**
**QPRT**	12.399	4.475	31.377	5.391	28.260±3.771 **↑compared to HFA C**	18.734±3.004 **↑compared to HFA IFN**
**ACMSD**	4.167	2.271	1.093	0.577	2.155±0.362 **↓compared to HFA C ↑compared to AA C**	1.082±0.472 **↓compared to Glioma C**

↑*denotes significantly higher expression;*

↓*denotes significantly lower expression; Relative expression values are shown in arbitrary units. Data presented as the means ±SEM, unless otherwise shown.*

IDO-1 expression in both IFN-γ stimulated AA and HFA was higher compared to when unstimulated. IDO-1 was expressed in all glioma samples when untreated and treated. IDO-2 expression was higher in HFA after stimulation with IFN-γ compared to untreated. IDO-2 was constitutively expressed by the vast majority (approximately 80%) of glioma samples analysed. In response to IFN-γ stimulation, all of glioma analysed demonstrated IDO-2 expression. The AA control time point sample for IDO-2 expression was omitted from analysis due to primer design issues. TDO-2 was constitutively expressed by the vast majority (approximately 89%) of gliomas analysed. TDO-2 was expressed by all of glioma analysed when stimulated with IFN-γ. TDO-2 was expressed both constitutively and following IFN-γ stimulation in HFA cells. Both AA samples (control and IFN-γ) for TDO-2 expression were omitted from analysis due to primer design issues. AFMID expression was lower in all cell cultures when stimulated compared to when unstimulated. The HFA control time point sample for KYNU expression was omitted from analysis due to primer design issues. AFMID and KMO were expressed by all cell types both constitutively and upon IFN-γ stimulation. KYNU was expressed by HFA when stimulated with IFN-γ. KAT-I and KAT-II expression was lower in HFA and AA cells when stimulated compared to when unstimulated. KAT-III expression was higher in HFA cells but lower in AA cells when stimulated with IFN-γ compared to when unstimulated. KAT-I, II and III were all expressed constitutively and following IFN-γ stimulation in both HFA and AA cells. No statistical significance was observed for 3-HAAO expression. All IFN-γ stimulated cultures showed higher 3-HAAO expression compared to when unstimulated. QPRT expression was lower when all cultures were stimulated compared to when unstimulated. HFA and AA- IFN-γ stimulated cultures showed lower ACMSD expression compared to when unstimulated. 3-HAAO, QPRT and ACMSD were all expressed constitutively and following IFN-γ stimulation in both HFA and AA cells.

### Quantification of KP metabolites determined by HPLC and GCMS

**Figure 5:**
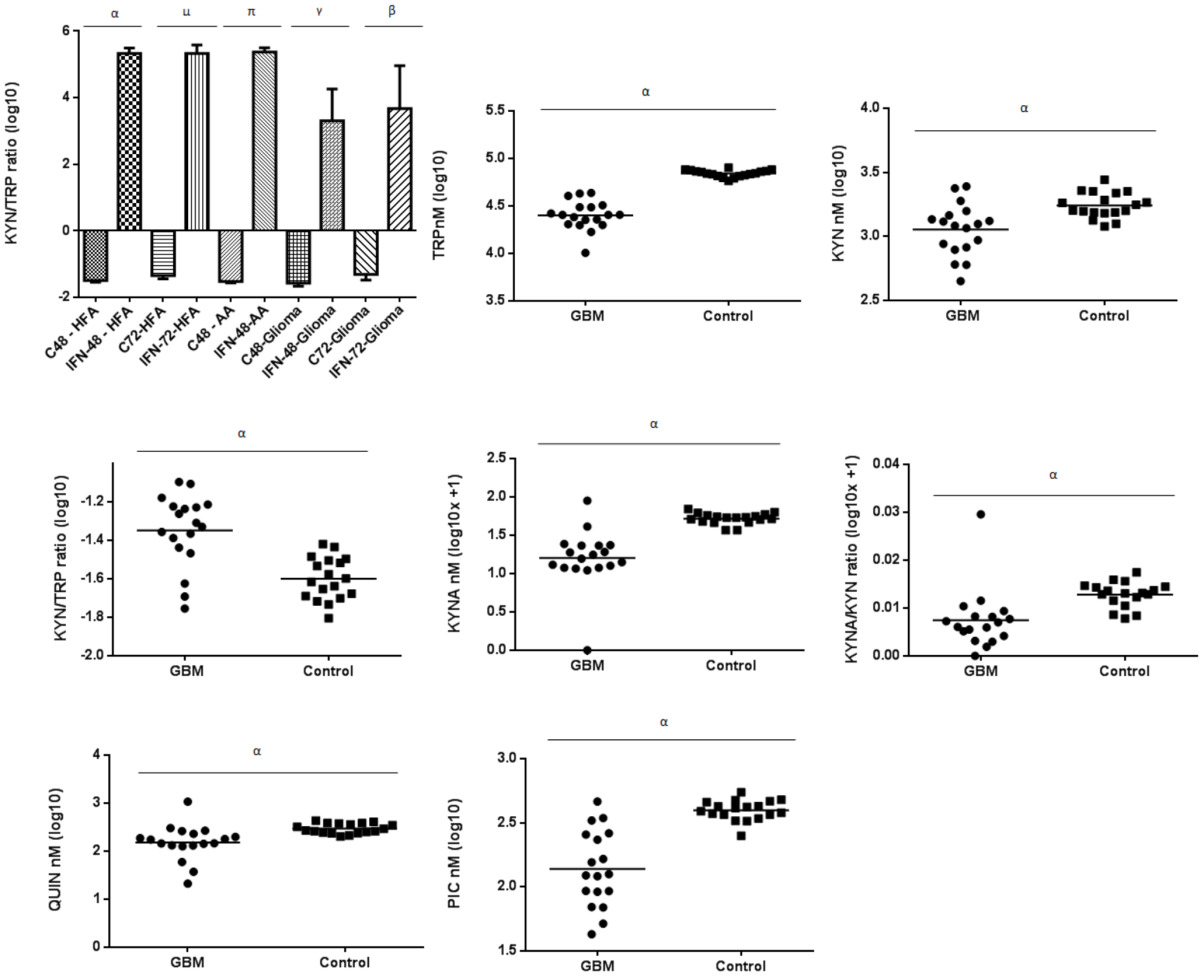
Analysis of the KYN/TRP ratio for IDO activity in culture supernatants of HFA, AA and glioma determined by HPLC and analysis of plasma Tryptophan (TRP), Kynurenine (KYN), Quinolinic acid (QUIN), Picolinic acid (PIC) and Kynurenic acid (KYNA) concentrations and the Kynurenine (KYN)/Tryptophan (TRP) and KYNA/KYN ratios from normal healthy controls (n = 18) versus GBM patients (n = 18). The graph and dot plots indicate Log10 of the quantified values unless otherwise shown. Glioma cell cultures were untreated (n = 7) and treated with IFN-γ (n = 6) for 48 hours and untreated (n = 6) and treated (n = 6) with IFN-γ for 72 hours. HFA cell cultures were untreated (n = 10) and treated with IFN-γ (n = 9) for 48 hours and untreated (n = 5) and treated with IFN-γ for 72 hours (n = 5). AA cell cultures were untreated (n = 3) and treated with IFN-γ (n = 3) for 48 hours. IDO activity in HFA cell cultures after 48 and 72 hours of IFN-γ stimulation compared to untreated cultures (^αµ^p<0.001). IDO activity in AA cell cultures after 48 hours of IFN-γ stimulation compared to untreated cultures (^π^p<0.001). IDO activity in gliomas after 48 and 72 hours of IFN-γ stimulation compared to untreated cultures (^γβ^p<0.05). No other statistical significance was observed. The GBM plasma samples consisted of: recurrent GBM (n = 9), secondary GBM (n = 5), primary GBM (n = 1), PNET GBM (n = 2) and unclassified GBM (n = 1). Each dot represents data derived from 1 sample. Horizontal line in each group designates the mean value. TRP concentrations in GBM patients compared to that of the control group (^α^p<0.0001). KYN concentrations in GBM compared to Control (^α^p = 0.0005). The KYN/TRP ratio for IDO activity in GBM compared to Control (^α^p<0.0001). KYNA concentrations in GBM patients compared to that of the control group (^α^p<0.0001). KYNA/KYN ratio in GBM compared to the control group (^α^p = 0.001). The GBM plasma samples analysed for QUIN and PIC concentrations consisted of: recurrent GBM (n = 7), secondary GBM (n = 3), PNET GBM (n = 2) and unclassified GBM (n = 1). QUIN concentrations in GBM compared to controls (^α^p = 0.0014). PIC concentrations in GBM compared to the control group (^α^p<0.0001).

KP metabolites were quantified in the cell culture supernatants of cultured HFA, AA and cultured glioma patient samples determined by HPLC and GCMS (Figure 4B and Figure 5). These results are summarised in Table 2. KYNA was produced constitutively and following IFN-γ stimulation for 48 and 72 hours in all cell cultures. All cell cultures also produced higher KYNA concentrations after stimulation, although this did not reach significance. No statistical significance was observed for QUIN and PIC concentrations in all cells. TRP was catabolised and not produced by all cell cultures, as the levels of TRP were lower than initially quantified in the culture medium (exhausting TRP in the culture medium). HFA cell cultures consumed KYN from the growing medium when untreated for 48 hours. All cell types in culture produced very little KYN constitutively at all time points. All cell types also catabolised TRP constitutively and following IFN-γ stimulation.

**Table 2:** Summary of KP metabolite levels in the cell culture supernatants of glioma, HFA and AA cells using HPLC and GCMS.

KP metabolite levels (nM/mg protein)	C48 HFA	IFN-48-HFA	C72- HFA	IFN-72-HFA	C48-AA	IFN-48-AA	C48-Glioma	IFN-48-Glioma	C72-Glioma	IFN-72-Glioma
**KYNA/KYN ratio**	0.135±0.010	0.004±0.001 **↓compared to C48 HFA**	0.135±0.018	0.006±0.000 **↓compared to C72 HFA**	0.126±0.009	0.004±2.052e-005 **↓compared to C48 AA**	0.109±0.010	0.003±0.000 **↓compared to C48 Glioma**	0.080±0.017	0.025±0.020 **↓compared to C72 Glioma**
**KYNA**	87.14±67.05	267.5±126.5 **↑compared to C48 HFA**	-	-	-	-	-	-	-	-
**KYN**	-785.6±593.5	395097±175354 **↑compared C48 HFA**	-	-	77.11±352.6	248527±78642 **↑compared to C48 AA**	1282±724.1	393739±110090 **↑compared to C48 Glioma**	6999±6714	337214±108065 **↑compared to C72 Glioma**
**TRP**	-	-	-	-	-21961±5338	(-258896±81590 **↑compared to C48 AA**	-16423±40388	-379302±100809 **↑compared to C48 Glioma**	-35390±36680	-356702±127988 **↑compared to C72 Glioma**
**KYN/TRP ratio**	0.034±0.005	407035±181942 **↑compared to C48 HFA**	0.05±0.012	538770±419926 **↑compared to C72 HFA**	0.030±0.003	255976±82149 **↑compared to C48 AA**	0.032±0.010	135073±65531 **↑compared to C48 Glioma**	0.071±0.026	290577±107924 **↑compared to C72 Glioma**

↑*denotes significantly higher;*

↓*denotes significantly lower; Note: only those values which were significant are presented in the table; Data presented as the means ±SEM, unless otherwise shown; KYNA/KYN and KYN/TRP ratio levels are expressed in arbitrary units.*

KP metabolite levels were quantified in the plasma of GBM patients (n = 18) versus healthy control subjects (n = 18) (Figure 5). These results are summarised in Table 3.

**Table 3:** Summary of KP metabolite levels in the plasma of GBM patients and healthy control subjects using HPLC and GCMS.

KP metabolite levels (nM)	Control plasma (n = 18)	GBM patient plasma (n = 18)
**TRP**	69739±1376	26628±2092 **↓compared to control plasma**
**KYN**	1791±98.28	1247±133.3 **↓compared to control plasma**
**KYN/TRP ratio**	0.026±0.002	0.049±0.004 **↑compared to control plasma**
**KYNA**	52.89±1.969	21.30±4.448 **↓compared to control plasma**
**KYNA/KYN ratio**	1.030±0.002	1.018±0.004 **↓compared to control plasma**
**QUIN**	304.9±17.28	212.5±54.01 **↓compared to control plasma**
**PIC**	401.3±16.51	171.6±27.80 **↓compared to control plasma**

↑*denotes significantly higher;*

↓*denotes significantly lower; Data presented as the means ±SEM; KYNA/KYN and KYN/TRP ratio levels are expressed in arbitrary units.*

## Discussion

Herein, we are the first to fully characterise the KP in cultured human primary glioma specimens and GBM patient plasma versus controls. It has been demonstrated that tryptophan degradation is induced by IFN-γ in a number of cells and cell lines (SK-N-SH, neuroblastoma; T 24, J 82, bladder carcinoma; A 431, epidermoid carcinoma; normal dermal fibroblasts, U 138 MG, glioblastoma; SK-HEP-1, hepatoma; A 549, lung carcinoma; A 498, kidney carcinoma) [21]. Analysis of tumor tryptophan uptake in 40 patients with primary brain tumors and analysis of tryptophan metabolism quantified in 23 patients revealed that gliomas and glioneuronal tumors have an elevated tryptophan uptake and catabolism *in vivo* [28]. It is also known that IDO-1 activity in human glial and malignant glioma cell lines is increased by IFN-γ [29]. Miyazaki et al., [20] demonstrated that in several cultured human malignant glioma cell lines, exposure to IFN-γ steeply decreased the level of TRP in the culture medium concomitant with greatly increased IDO-1 expression of the cells. Guillemin et al., [30] demonstrated that both human primary neurons and the SK-N-SH neuroblastoma cell line increased TRP catabolism concomitant with greatly increased KYN production following IFN-γ stimulation for 24 hours compared to untreated cell cultures.

Our data complement and extend these findings, showing that KYN production and TRP catabolism in glioma cell cultures was significantly higher after 48 and 72 hours of IFN-γ stimulation compared to untreated cultures. The increase in TRP catabolism with concomitant increases in KYN concentrations undoubtedly translated into higher KYN/TRP ratios, as our findings showed that KP activation (KYN/TRP ratio) was significantly increased in glioma after stimulating with IFN-γ for 48 and 72 hours compared to untreated cells. The TRP breakdown index has been used in many studies to indirectly indicate the sum of the activities of TDO-2, IDO-1 and IDO-2, and is calculated by the KYN/TRP ratio [31]. The increase in KP activity upon IFN-γ stimulation in cultured glioma cells is of significance, as KP activity in brain tumors *in vivo* is likely to be triggered when IFN-γ is produced from the surrounding activated T-cells and/or microglia and neurons.

In a study conducted by Uyttenhove et al. [15], immunohistochemical constitutive IDO-1 expression was observed in cases of prostatic, colorectal, pancreatic, cervical, and in nine of ten GBM carcinomas, in addition to several other tumor types. IDO-1 expression with IFN-γ stimulation has also been observed in colorectal [32] and lung cancers [17]. Similarly, other investigators showed that a variety of cancer cell types including, ovarian [18], and neuroblastoma [30] and GBM cell lines [21] over-expressed the IDO-1 enzyme, either constitutively or following IFN-γ stimulation when compared to normal cells. Our qRT-PCR analyses further substantiate and corroborate these findings revealing that IDO-1 expression in glioma cells was significantly up-regulated after 24 hours of IFN-γ stimulation compared to unstimulated glioma cells. In addition, all glioma samples demonstrated constitutive IDO-1 expression. Taken together, over-activation (high KYN/TRP) of the KP, up-regulated IDO-1 expression, and enhanced TRP catabolism in glioma cells in response to IFN-γ and the IDO-1 over- expression in glioma cells is likely to play a role in mediating brain tumor immune evasion via inhibiting tumor-specific immunity and driving malignant progression. IDO-1 up-regulation and induction in glioma cells may function to deplete TRP from the tissue microenvironment and accumulate KP-derived toxic metabolites, which may ultimately suppress T-cell proliferation [33]–[35].

The significantly higher IDO-2 expression in IFN-γ stimulated glioma cells compared to unstimulated, may suggest that IDO-2, like IDO-1 can be potentiated by IFN-γ. The significant increase in both IDO-1 and IDO-2 expression in response to IFN-γ stimulation may suggest that both enzymes could contribute to tumor progression by inhibiting specific tumor immunity. The significant increase in constitutive IDO-2 expression in glioma cells compared to constitutive expression in HFA further corroborates this hypothesis. These findings may also indicate that pro-inflammatory cytokines/stimuli could play a role in IDO-1/2 induction in glioma patients.

We also showed that TDO-2 was constitutively expressed by the vast majority (approximately 89%) of glioma samples analysed. These findings parallel recently published data showing that TDO-2 is frequently activated and constitutively expressed in human glioma cells, A172, LN-18, U87 and T323 human glioma-initiating cells [23].

Expression of KYNU and KMO mRNA was shown to be present at low levels in both the IFN-γ stimulated and unstimulated in the SK-N-SH neuroblastoma cell line [30]. We observed both KMO and KYNU expression to be significantly up-regulated in IFN-γ stimulated glioma compared to unstimulated glioma cells. This may reflect the dysregulated state of glioma cells in response to pro-inflammatory stimuli in the tumor microenvironment. Up-regulation of both KMO and KYNU in glioma may lead to increased production of 3-HK and 3-HAA, which may contribute to creating an immunosuppressive micromilieu by accumulating tryptophan toxic metabolites. However in contrast, both KYNU and KMO expression was significantly lower in stimulated and unstimulated glioma cells compared to stimulated AA and unstimulated HFA, respectively.

The kynurenine aminotransferases (KAT-I, -II, and -III) are responsible for synthesising the neuroprotectant KYNA [36]. Very little is known about the specific biochemical activity of KAT-III, moreover it may vary between different species [37]. Within the CNS, most KYNA is produced by astrocytes, neurons, and oligodendrocytes [38], [39]. KAT-I and KAT-II enzyme expression is well correlated with the ability of primary astrocytes to produce KYNA from KYN [40]. Both IFN-γ stimulated and unstimulated SK-N-SH cells showed expression of both KAT-I and KAT-II [30]. This is consistent with our results, showing that glioma cells expressed both KAT-I and KAT-II constitutively and upon IFN-γ stimulation. KAT-III was also expressed constitutively and following IFN-γ stimulation in glioma cells. Further, the down-regulation of all KATs in glioma compared to HFA in both inflammatory and constitutive conditions and the down-regulation of KAT-I in glioma cells in response to IFN-γ coincides with our KYNA/KYN ratio results.

The KYNA/KYN ratio is sometimes termed the neuroprotective ratio and has been extensively used as an index of neuroprotection in a variety of studies [41], [42]. A number of studies have found a shift in balance between neurotoxic and neuroprotective metabolites of KYN, in depressed patients [43], renal carcinoma patients [41], and schizophrenic patients [42]. IFN-γ-mediated KAT-I down-regulation in glioma and the down-regulation of all KATs in glioma compared to HFA may subsequently function to have a reduced capacity to produce the neuroprotective metabolite KYNA at the site of inflammation. However, although glioma cells did not show any significant reduction in KYNA production either when compared to controls or when stimulated, they did demonstrate a significantly lower neuroprotective ratio (KYNA/KYN) following IFN-γ stimulation. This implies that the metabolism of KYN is preferentially directed towards the intermediate metabolites of the KP and subsequently towards the NAD^+^ pathway under inflammatory conditions. This may further support the hypothesis that there is a disturbance in the KP in glioma in response to inflammatory stimuli, skewing away from the KYNA side branch. However, future studies will require the quantification of 3-HAA, 3-HK and NAD^+^ to determine whether there is subsequent formation and accumulation of 3-HAA, 3-HK and NAD^+^ synthesis.

The conversion of 3-HAA to the unstable intermediate, 2-amino-3-carboxymuconate semialdehyde (ACMS), involves the enzyme 3-hydroxyanthranilate 3,4-dioxygenase (3-HAAO) [44]. 3-HAAO and quinolinate phosphoribosyltransferase (QPRT) mRNA has been shown to be expressed constitutively and after IFN-γ stimulation in the neuroblastoma cell line, SK-N-SH, with both enzyme levels being slightly higher in SK-N-SH compared with controls, primary neurons, although not significantly [30]. Our results are partly consistent with these findings showing that both 3-HAAO and QPRT were expressed in all glioma samples constitutively and upon IFN-γ stimulation. QPRT expression was significantly up-regulated in stimulated and unstimulated glioma compared to stimulated and unstimulated HFA, respectively, confirming the NAD^+^ hypothesis.

PIC has been suggested to possess neuroprotective and a number of anti-proliferative effects within the CNS [30], [45]. The literature surrounding PIC is limited, and its exact endogenous function is not known [45]. The activity of ACMS decarboxylase (ACMSD) determines whether the metabolites in the KP are converted to QUIN for NAD^+^ biosynthesis or to PIC [46]. Ikeda et al., [47] first presented evidence showing that the activity of ACMSD is inversely proportional to the amount of NAD^+^ synthesized from tryptophan. ACMSD is therefore a key enzyme directing KP metabolism towards PIC production [48].

Our results suggest that QPRT degrades QUIN more readily in glioma compared to in controls, which may give rise to high levels of NAD^+^. Up-regulation of QPRT expression compared to HFA constitutively and in response to inflammatory stimuli (IFN-γ) may be a mechanism conferring tumor survival advantages, as the glioma may have a greater capacity to produce NAD^+^ in times of inflammatory stress. The increased availability of NAD^+^ may provide an improved supply of substrate to the DNA repair associated enzyme poly(ADP-ribose) polymerase (PARP) [49], [50], assisting in tumor cell DNA replication and repair and promote the maintenance of high metabolic activity, ultimately promoting tumor cell viability and proliferation [51]. Analysis of NAD^+^ concentrations in the glioma culture medium may give further insight into whether QUIN is catabolised more rapidly in glioma than in controls. Interestingly, a recent study indicates the specific capability of glioma cells to use QUIN as an NAD^+^ precursor, showing that neoplastic transformation in astrocytes is associated with a QPRT-mediated switch in NAD^+^ metabolism by exploiting microglia-derived QUIN as an alternative source of replenishing intracellular NAD^+^ pools [52].

Down-regulation of ACMSD in stimulated glioma cells compared to unstimulated glioma cells in the present study may further direct KP metabolism away from the metabolite PIC and towards QUIN and NAD^+^ production under conditions of inflammatory stress in the tumor microenvironment. These results are in contrast to that observed with the neuroblastoma cell line, where no ACMSD was detected by the neuroblastoma cell line [30], possibly due to less sensitive methods of detection. Fig. S1 displays a schematic diagram of the alterations in KP metabolism in cultured human primary glioma.

A study conducted by Opitz et al. [23], demonstrated TRP concentrations to be lower in the serum of patients with glioma, accompanied with no significant increase in KYN levels. Our data complement the above findings, showing that TRP concentrations were significantly lower in GBM patients compared to controls, accompanied with no significant increase in KYN levels. The higher KYN/TRP ratio in GBM patient plasma compared to controls indicates KP over-activation. These results may support a role for KP hyper-activation in tumor development and progression [51]. The most important consequence of a dramatic decline in TRP levels and higher K/T ratios is likely to be immunosuppression. These data further corroborate our *in vitro* results, showing higher KP activation and TRP degradation in glioma when stimulated with IFN-γ compared to unstimulated. KP up-regulation and induction in glioma may function to inhibit tumor-specific immunity through the depletion of tryptophan from the tumor microenvironment, which may ultimately suppress T-cell proliferation [33]. High KP activation may also lead to the accumulation of the later metabolites, 3-HK and 3-HAA.

The decrease in PIC levels in GBM patients may suggest that the KP is skewing towards neurotoxicity and away from neuroprotection. The significantly lower production of QUIN in GBM patient plasma compared to controls may suggest that the resulting neurotoxic burden on brain cells is limited. However, at this stage, this unexpected outcome is speculated to reflect that QUIN may be catabolised in GBM patients at a higher rate compared with controls, which may give rise to higher NAD^+^ levels. Further, an important point to note is that due to the plasma samples being taken only at one time point, it is undetermined at this stage whether low QUIN levels appear transiently or are maintained throughout the disease. Therefore, the speculation that QUIN accumulation may underly GBM tumorigenicity is neither supported nor rejected.

The significantly lower KYNA production and KYNA/KYN ratio in GBM patient plasma compared to controls, may suggest that the metabolism of KYN is preferentially directed into the neurotoxic QUIN pathway. These results are further supported by the significant reduction in PIC concentrations in GBM patients compared to controls in the current study. Both reduced KYNA and PIC production suggest that both neuroprotective branches of the KP are impaired. These data may represent a shunting of tryptophan away from the KYNA branch and towards the preferential accumulation of the intermediate metabolites and towards the neurotoxic metabolite and subsequently NAD^+^.

TRP and KYN readily cross the blood-brain barrier unlike KYNA and QUIN [53]. Therefore, a limitation of this study is the use of peripheral blood samples. Analysis of patient CSF will more accurately reflect alterations in brain tumor KP metabolism. The CSF bathes the tissues of the CNS [54], which may enable the direct secretion of brain tumor-derived KP metabolites into the CSF [55].

In conclusion, our data implicates the involvement of the KP in GBM pathophysiology and highlights specific KP metabolites and enzymes as highly attractive targets to evaluate in brain tumor biology. Together these characteristics may ultimately contribute to brain tumor persistence by: (1) mediating brain tumor immune evasion; (2) skewing the KP away from the neuroprotective branches of the KP; (3) preferentially accumulating the intermediate metabolites, 3-HK and 3-HAA which are T-cell apoptotic; and (4) having a greater capacity to produce NAD^+^ thereby aiding in the maintenance of cellular energy (ATP) metabolism and proliferation. Collectively, our data provide the foundation and rationale for further investigation into determining the potential therapeutic effects of KYNU and KMO pharmacological inhibitors in brain tumors. Inhibiting these enzymes may represent novel therapeutic strategies in glioma therapy. This is a particular need, since a recent study found that IDO-1 expression was paradoxically upregulated in human cancers from 1-D-MT, a compound currently used in a phase 2 clinical study in patients with metastatic breast cancer [56].

## Materials and Methods

### Ethics Statment

Approval for this study was obtained from the Human Research Ethics Committees of the University of New South Wales, St Vincent's Hospital and Macquarie University. Written informed consent was obtained from the participants. The study methodologies conformed to the standards set by the Declaration of Helsinki.

### Tumor Sampling and Data Collection

Glioma samples were collected from patients who underwent surgery at the Centre for Mininally Invasive Neurosurgery, Prince of Wales Private Hospital, NSW, Australia. The surrounding ‘healthy’ brain tissue was also acquired from patients during surgery for their tumor. A portion of the surgically resected brain tumor and the surrounding tissue sample was snap-frozen in liquid nitrogen immediately and stored at -80°C for prolonged storage for future use. The other portion was used for isolating glioma cells in cell culture. Brain tumor samples were graded according to the WHO criteria by experienced neuropathologists. Clinical information collected included patient age, gender and tumor type.

GBM (n = 6), OII (n = 1), AII (n = 1) and one unclassified tumor (n = 1) tissue samples were cultured to purity for analysis in GC/MS and HPLC. Of the six GBM patients, 4 were male and 2 were female ranging from 23 to 48 years of age with a mean age of 34 years. One GBM patient age was unidentified. The one OII patient was a female of 45 years of age. The one AII patient was a female of 28 years of age. Clinical data including the pathological verification of tumor type was unable to be obtained for 1 tumor and this was termed *‘Unclassified tumor’*. Human foetal (n = 10) and human adult (n = 3) brain tissue samples were cultured for the development of HFA and AA for analysis in qRT-PCR, HPLC and GC/MS. The three adult brain specimens which were used in qRT-PCR, GC/MS and HPLC were derived from 2 females and 1 male ranging from 10 to 61 years of age, with a mean age of 39.67 years. The foetal brain tissue used in GC/MS and HPLC were derived from 17-week-old (n = 3), 18-week-old (n = 2), 19-week-old (n = 4) and 20-week-old (n = 1) foetuses. GBM (n = 5), AII (n = 2), OII (n = 2) and one unclassified tumor (n = 1) tissue specimens were cultured to purity for analysis in qRT-PCR. Of the five GBM patients, 3 were male and 2 were female ranging from 23 to 46 years of age with a mean age of 33.6 years. The AII patients consisted of 2 females, 25 and 28 years of age with a mean age of 26.5 years. The OII patients consisted of 1 female and 1 male, 45 and 32 years of age, respectively. Clinical data including the pathological verification of tumor type was unable to be obtained for 1 tumor and this was termed *‘Unclassified tumor’*. Not all the tumors listed in this table were included in data analysis for qRT-PCR, as they were omitted due to primer design issues. The foetal brain tissue used in qRT-PCR were derived from 17-week-old (n = 3), 18-week-old (n = 1), and 19-week-old (n = 6) foetuses.

### Blood and Data Collection

Blood was collected pre-operatively from brain tumor patients who underwent surgery at the Centre for Minimally Invasive Neurosurgery, Prince of Wales Hospital, Sydney, Australia. Blood was collected by either the nurses or the anaesthetist at the time of surgery and stored in lithium heparin or EDTA vacutainer venous blood collection tubes (BD Biosciences). Blood samples were separated by centrifugation (1,600 g, 15 min). The resulting plasma was carefully removed and aliquoted into separate microcentrifuge tubes, which were then stored at −80^o^c until GC/MS and HPLC assays was performed. A proportion of plasma samples were obtained from the Steve and Lynette Waugh Brain Tumor Bank provided by Dr Kerrie McDonald. The plasma from 18 sex-matched and approximately age-matched healthy patients was used as the control samples. Of the plasma obtained from GBM patients (n = 18), 13 were male and 5 were female ranging from 22 to 76 years of age, with a mean age of 49.67 years. Of the plasma obtained from healthy patients (n = 18), 13 were male and 5 were female, ranging from 23 to 70 years of age, with a mean age of 47.72 years.

### Human primary glioma cell culture

Human glioma cells were isolated from surgically resected tissue from patients diagnosed with a glioma. Surgical tumor tissue specimens were obtained within 1 hour after surgical removal and were washed twice with Dulbecco's phosphphate buffered saline (DPBS) (Invitrogen, Australia) to remove contaminating blood. Visible blood vessels were aseptically removed with sterile scissors. The tissue was then diced into <1 mm^3^ fragments using sterile scissors. The minced tissue was transferred into a 50 ml falcon tube containing an enzyme cocktail mixture for further dissociation of the tissue. The enzyme cocktail mixture contained collagenase type IV (0.03%–0.1%) (Invitrogen, Australia) prepared in HBSS (Invitrogen, Australia). This was incubated at 37°C for 30 min with occasional agitation of the falcon tube. After 30 mins in the incubator, the tumor slurry was then triturated with a syringe attached to a mixing cannula. The volume of the tube was then maximised with DPBS (Invitrogen, Australia) and centrifuged at 200 g for 10 min. The supernatant was then aspirated and the cell pellet resuspended in DMEM/F12/RPMI (1∶1) media (Invitrogen) supplemented with 10% FBS, 1% L-glutamine, 1% Glucose and 1% antibiotic/antimycotic. Trituration of the mixture resulted in a homogenous cell suspension. The cell suspension was then transferred to a culture flask and incubated at 37°C in a humidified atmosphere containing 95% air and 5% carbon dioxide. The cultures were carefully inspected the following day after plating and the debris from red blood cells and unattached fragments were rigorously washed off with DPBS followed by the addition of fresh medium to each flask. Medium changes were then biweekly until cells grew to confluence. Tumor cell cultures reached confluency within 2–3 weeks. After cells reached confluence, they were subjected to successive trypsinisations (2–3 passages) with TrypLE (Invitrogen, Australia) for the removal of contaminating cells. The cells which were passaged were replated at low cell density. Medium changes were then triweekly until cells grew to confluence. Cultures were maintained for up to 5 weeks in total. After confirmation that the cells were of astrocytic origin using an astrocytic marker (GFAP), the cells were detached with TrypLE express, resuspended in medium and plated in 6-well culture plates for experimentation.

### Human primary foetal astrocyte and adult astrocyte cell culture

Human foetal brain tissue was obtained from 17 to 20-week-old foetuses collected after therapeutic termination following informed consent. These were used to isolate pure cultures of human foetal astrocytes (HFA) from resected brain tissue from 10 separate human foetuses. Three normal brain tissue specimens were acquired from consented adult patients during surgery for their tumor. These were used to develop pure cultures of AA from 3 different patients.

For the preparation of HFA and AA cultures, a small piece of brain was washed twice in DPBS to remove contaminating blood. Visible blood vessels were aseptically removed with sterile scissors. The tissue was placed in a 50 mL falcon tube containing 20 mL RPMI medium supplemented with 10% FBS, 1% L-glutamine, 1% Glucose and 1% antibiotic/antimycotic. Repeated trituration of the mixture with a mixing cannula attached to a syringe resulted in a homogenous cell suspension. This method was referred to as mechanical dispersion, which is dissimilar to enzymatic dissociation. The cell suspension was then transferred to a culture flask and incubated at 37°C in a humidified atmosphere containing 95% air and 5% carbon dioxide. The cultures were carefully inspected the following day after plating and the debris from red blood cells and unattached fragments are rigorously washed off with DPBS followed by the addition of fresh medium to each flask. Medium changes were then biweekly until cells grew to confluence. After confluence, the methods for astrocytic purification were similar to that followed for glioma cell isolation. Astrocyte cultures became confluent after 10–15 days. After cells reached confluence, they were subjected to successive trypsinisations (2–3 passages) with TrypLE for the removal of contaminating cells. Medium changes were then triweekly until cells grew to confluence. Cultures were maintained for up to 5 weeks in total. After confirmation the cells were of ‘astrocytic lineage’, the cells were detached with TrypLE express, resuspended in medium and plated in new flasks for experimentation.

### Cell culture purification

Viable tumor cells attach within the first several days of culture, whereas the majority of glia do not [57]. Normal brain cells, such as microglia, fibroblasts, endothelial cells, oligodendrocytes, and neurons may initially attach, although generally in small proportions [57]. However, their numbers usually decline rapidly and eventually they will be eliminated from culture because of their slow growth and repeated passaging/medium changes [57]. The most efficient method in this study at minimising microglia and neurons was the subculturing method, which consisted of repeated passaging. A number of studies have also utilised the increased adherence properties of microglia on uncoated tissue culture plastic to separate the different cell types [27] and the present study found that subculturing by detaching a small proportion of cells in the monolayer resulted in the majority of astrocytes being detached, while leaving the microglia behind.

### Astrocyte and Glioma Culture Treatments

Prior to receiving any treatment, pure astrocyte and glioma cell cultures were allowed to equilibrate for 24 hours at 37°C in a humidified atmosphere containing 95% air/5% CO_2_. All cultures were either untreated (control) or treated with IFN-γ (100 IU/mL) for each experiment. Following the addition of IFN-γ, cultures were incubated for 48 and 72 hours before the collection of supernatant for GCMS/HPLC analysis and incubated for 24 hours before cell lysis for RNA extraction.

### Immunocytochemistry (ICC)

The expression of astrocytic and oligodendroglial lineage markers for glioma tumor cells, HFA and AA were verified by immunostaining of the cells with the astrocytic marker GFAP. Determination of the presence of microglia was performed and contamination was excluded by showing lack of binding of the microglia-specific antibodies, CD68 and CD11b in the majority of cultures. This was performed prior to the commencement of any experimentation. However, not all cultures were confirmed for microglia presence. Only initial cultures were analysed.

After the purification steps in all cultures, pure cells were then grown in slide flasks (Nunc). When cultures reached 70% confluence, the media was removed and the cells were washed twice with DPBS prior to fixation. The cells were then fixed with methanol-acetone (1∶1) at −20°C for 10 min. Membranous permeabilization followed after two washings with ice-cold DPBS. Cells were immersed in 0.1% Triton-X in DPBS for 10 min at room temperature. Cells were then washed again with DPBS and incubated in 4% NGS in PBS for at least 30 min at 4°C. Following that, the cells were washed again and dried. A Dakocytomation pen (Dako) was then used to enclose the cells in separate wells. The primary antibodies (Table S2), diluted in 4% NGS, were applied. The following negative control was performed for each experiment: (i) incubation with only the secondary labeled antibodies. This was conducted to detect any non-specific binding with the secondary antibodies. The slides were returned back to the humidity chamber and incubated for another 1 hour. After the incubation period, the cells were again washed extensively with DPBS and dried. ProLong Gold Antifade Reagent with DAPI (Invitrogen) (for nuclear staining) was applied directly to fluorescently labeled cells. The cells were then cover-slipped and the edges were sealed with nail varnish. They were then incubated for 24 hours at room temperature in the dark before examination with the Leica SP5 confocal microscope equipped with MultiPhoton laser (Leica Microsystems, Australia).

### Total RNA Extraction

Total RNA was extracted from cultured cells using the PureLink RNA Mini Kit (Ambion, Life technologies, Australia) and an extra step involving DNase treatment (PureLink Dnase from Ambion, Life technologies) was conducted to remove contaminating DNA in the sample according to the manufacturer's protocol. This step involved On-column DNase treatment. The final step involved eluting RNA in 30 µl of UltraPure water. RNA quantity and purity was evaluated spectrophotometrically by determining the ratio of UV absorbance readings at 260 nm and 280 nm (A260/A280) with the NanoDrop 2000c Spectrophotometer (Thermo Scientific, Australia). The RNA Integrity Number (RIN) was confirmed by the Agilent 2100 electrophoresis (Agilent Technologies, Australia) Bioanalyzer by the Ramaciotti Centre (UNSW, Sydney, Australia). The RNA was then stored at −80°C until *cDNA synthesis*.

### cDNA Synthesis

1 µg of total RNA in a final volume of 20 µl was used for the synthesis of cDNA using the Superscript Vilo cDNA Synthesis Kit (Ambion, Life technologies, Australia) in accordance with the manufacturer's recommendations. The protocol for cDNA synthesis has been optimized for generating first-strand cDNA for use in two-step qRT-PCR. Briefly, for a single reaction, the following components were combined in a tube on ice: 4 ul of 5× VILO Reaction Mix, 2 µl of 10× SuperScript Enzyme Mix, 1 µg of RNA and DEPC-treated water to a final volume of 20 µl. A separate tube which was the No-RT reaction tube consisted of all of the above components but with omission of the Enzyme Mix. This was used as a control to confirm that no genomic DNA was present in the sample. The tube was gently mixed and then placed on a Mastercycler gradient PCR machine (Eppendorf, Australia) for the cDNA synthesis reaction with the following parameters: (1) incubation at 25°C for 10 minutes (2) incubation at 42°C for 60 minutes, and (3) termination of the reaction at 85°C at 5 minutes. The resulting cDNA was then stored at -80°C until further use.

### Design and Validation of Oligonucleotides for quantitative real-time polymerase chain reaction (qRT-PCR) amplification

Briefly, primers were obtained from several sources: designed by Dr. Fabrice Magnino (PCR/qPCR specialist, Integrated Sciences Pty Limited), a gift from collaborating researches, Primer Bank (http://pga.mgh.harvard.edu/primerbank/) or designed by using the Primer BLAST (web-based NCBI primer designing tool: http://www.ncbi.nlm.nih.gov/tools/primer-blast/) or the Primer3 designing programme (http://primer3.sourceforge.net/). All obtained sequences were run through “Amplify 3x” (http://engels.genetics.wisc.edu/amplify/) and “NetPrimer” (http://www.premierbiosoft.com/index.html) software and blasted with Primer BLAST for determination of exon/intron boundaries and evaluating the best potential outcome based on relative scores and secondary structures. The following parameters were used during the design: melting temperatures 58–61°C, primer lengths 20–24 bp and amplicon lengths 80–200 bp. 3 to 5 primer pairs were directed to locate on different exons or directly spanning exon-exon junctions of each cDNA. For each primer pair, reaction efficiency estimates were derived from a standard curve generated from a serial dilution of selected cDNA.

The primer sequence for hypoxanthine phosphoribosyltransferase (HPRT) and ACMSD transcripts were obtained from various already published studies. Briefly, primer optimisation for the HPRT and ACMSD transcripts was performed using a standard curve derived from serial dilutions (4 to 6) of human macrophage (for ACMSD) and HFA (for HPRT) cDNA (0.5 pg–50 ng). cDNA for the ACMSD transcript consisted of 4 serial dilutions ranging from 1.56 ng to 25 ng (2 times dilution factor). cDNA for the HPRT transcript consisted of 6 serial dilutions ranging from 0.5 pg–50 ng. The specificity of the PCR product and efficiency were then assessed. Specificity was verified by a single peak in melting curve analysis. The reaction efficiency was derived from the standard curve where the mean Ct values (of each dilution point) were plotted against the logarithm of the cDNA dilution factor. Each dilution was assayed in duplicate. The forward and reverse primer sequences and other primer parameters are given in Table S1.

### qRT-PCR reaction

The resulting cDNA from both the HFA and AA samples were separately ‘pooled’ together and these samples were then used as the representive ‘controls’ to compare against the brain tumor samples. Human macrophages (kindly prepared by Gloria Castellano Gonzalez) were used as a positive control for target gene expression. The qRT- PCR assay for unknown samples was performed simultaneously with positive control samples in the same plate. cDNA was diluted to a concentration of 2.5 ng/µl with RNase-free water. Briefly, 5 µl of the diluted synthesized cDNA together with the appropriate primers was added to 10 µl Express SYBR GreenER qPCR Supermix Universal (Life technologies) to a total volume of 20 ul. Primers were used at 300 nM final concentration. A PCR reaction mastermix was prepared for each primer before dispensing into 96× PCR plate cells. No-template control (NTC) reactions were also prepared for each gene. These consisted of all reaction components except for cDNA, which was replaced by 5 ul of DNase-free water. The cDNA of the No-RT reaction samples were also used to confirm that no genomic DNA was present in the samples. This was only performed in initial pilot studies where only 4–6 samples were initially tested for the presence of genomic DNA after DNase treatment of the samples. qRT-PCR was carried out using a Mx3500P Real-Time PCR system (Stratagene, NSW, Australia). The cycling parameters for all genes were the following: initial denaturation at 95°C for 3 min, then 40 cycles of 95°C for 20 s, and 60°C for 20 s. The annealing temperatures were optimised however for each primer. All transcripts were measured in each unknown sample in duplicates. Target gene expression values were normalised to an endogenous reference gene (HPRT) and were expressed relative to a calibrator sample (macrophages-positive control), determined by the equation below. The equation uses Efficiency (E) of the primers: 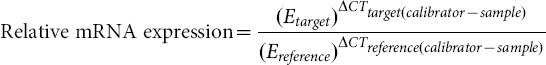



### Gas chromatography/mass spectrometry (GC/MS)

GC/MS was performed to determine the levels of QUIN and PIC in the supernatant of growing cells in culture stimulated with and without IFN-γ (100 U/ml) for 48 and 72 hours. Changes in the concentrations of QUIN and PIC in the culture medium were calculated using the following formula: (concentrations in the cell culture supernatant – concentrations initially present in the culture medium before adding to the cells). GC/MS was also performed to determine the levels of QUIN and PIC in the plasma of patients with glioma. The GC/MS method used for the analysis of PIC and QUIN has been described previously [58]. Briefly, the internal standards used were Picolinic acid conjugated with deuterium D4-PIC and Quinolinic acid conjugated with deuterium D3-QUIN. For both culture supernatants and plasma, the protein was precipitated by the addition of an equal volume of 10% TCA (1∶1) and centrifuged at 1000 rpm for 5 min. For the derivatisation procedure, standards and sample solutions (10–50 µL) were then transferred to glass tissue culture vials (100×10 mm) with the addition of 10 µl PIC and 20 µl QUIN internal standards and then evaporated to dryness using the SpeedVac. 60 µl of TFAA and 60 µl of HFP were then added to the residues. The glass vials were then capped and sealed immediately and heated at 60°C for 30 min to produce the hexafluoroisopropyl ester of the respective acids. The ester products were then dissolved in 180–250 µl of toluene, washed with 5% sodium bicarbonate (1 ml) and water (1 ml), dried over anhydrous sodium sulfate (approx 500 mg), and transferred to autosampler vials prior to injection (1 µL) into the GC/MS via 7683 Autosampler (Agilent). The final concentrations of unknowns were calculated by interpolation of the standard curve.

### High Performance Liquid Chromatography (HPLC)

HPLC was performed to determine the levels of TRP, KYN and KYNA in the supernatant of growing cells in culture which were unstimulated and stimulated with IFN-γ (100 U/ml) for 48 and 72 hours. Changes in the concentrations of TRP, KYN and KYNA in the culture medium were calculated using the following formula: [concentrations in the cell culture supernatant –concentrations initially present in the culture medium before it was added to the cells]. HPLC was also performed to determine the levels of TRP, KYN and KYNA in the plasma of GBM patients.

TRP and KYN were assayed concurrently using the Agilent 1200 series HPLC system complete with fluorescence and multi-wavelength detector in accordance to a method described previously [30] with slight modification. KYNA was also assayed by the Agilent 1200 series HPLC system, equipped with a fluorescence detector as outlined in [59] with minor changes.

The HPLC analysis was controlled using ChemStation software (Agilent Technologies, Australia). The standards and samples were applied to an Agilent Zorbax Eclipse XDB-C18 (5 µm, 150×4.6 mm i.d.) column (Agilent Technologies, Australia) at an injection volume of 30 µl. The analysis was carried out at a flow rate of 1 ml/min, at 22°C. The mobile phase consisted of 0.1 M ammonia acetate, at pH 4.65 and was filtered through a filtering system prior to usage and pumped isocratically at a flow rate of 0.8 ml/min. TRP was measured using a fluorescence detection at an excitation wavelength of 254 nm and an emission wavelength of 404 nm while KYN was detected using a multi-wavelength detection set at 365 nm. KYNA was eluted isocratically at a flow rate of 0.75 ml/min with a mobile phase consisting of 50 mM sodium acetate with 0.25 M of zinc acetate and 2.25% (v/v) acetonitrile. KYNA is detected using a fluorescence detector at an excitation wavelength of 344 nm and an emission wavelength of 388 nm. The final concentrations of unknowns were calculated by interpolation of the standard curve.

### Protein Assay

QUIN, PIC, TRP, KYN and KYNA concentrations analysed in the cell culture supernatant of cells were adjusted for variations in cell number by measuring the total protein per culture well using the Pierce BCA Protein Assay Kit (Thermo Scientific, Australia). The metabolite concentrations were calculated as a ratio of the total amount of protein in the well, expressed as nM/mg total protein. The quantification of protein was carried out according to the manufacturer's protocol.

### Statistical Analysis

The graphical data plots are expressed as the logarithm (log10 or log10 (x+1)) of raw values, unless otherwise shown. Log10 (x+1) was used for the values which contained a “0” (no enzyme expression) value. The data reported in the results section are expressed as the raw data values before transforming the data to a logarithm. Relative expression values for the qRT-PCR data are shown in arbitrary units. Data will be presented as the means ± the standard error of the mean (SEM) in the main text. The exception to this is the data obtained for HFA and AA in the qRT-PCR section, where only the mean is shown as no SEM could be obtained due to sample pooling. Parametric vaiables were compared by applying the one-way analysis of variance ANOVA *Tukey's multiple comparison test* for multi-group analysis and the Student's *t* test (one-tailed and unpaired) was used for 2 group analysis. The one-sample *t* test was used for comparing GBM to the pooled HFA and AA. For the analysis of KYN concentrations and TRP catabolism in GBM, HFA and AA culture supernatants using HPLC, separate Student's *t* tests were performed and not ANOVA. All analyses were conducted using GraphPad Prism 5 (GraphPad software, San Diego, CA, USA)). Statistical significance was accepted at P<0.05.

## Supporting Information

Figure S1 **Schematic diagram of the alterations in KP metabolism in cultured human primary glioma.** Schematic diagram of the KP in cultured human primary glioma in the presence of IFN-γ. Arrows indicate significant metabolite over/under - production in response to IFN-γ stimulation for 48 and 72 hours compared to untreated. Arrows indicate significant enzyme up/down-regulation in response to IFN-γ stimulation for 24 hours compared to untreated. IFN-γ stimulation significantly potentiated the expression of IDO-1 IDO-2, KYNU, KMO and significantly down-regulated ACMSD and KAT-I expression. This significantly increased KP activity but significantly lowered the KYNA/KYN neuroprotective ratio.
(DOCX)

Table S1 **qRT- PCR forward and reverse primer sequences and other parameters.**
(DOCX)

Table S2 **Summary of primary and secondary antibody parameters used in ICC analysis.**
(DOCX)
