# Supplementary material for: Involvement of the Kynurenine Pathway in Human Glioma Pathophysiology
Source: PLoS One. 2014 Nov 21;9(11):e112945. doi: 10.1371/journal.pone.0112945 (PMC4240539; doi:10.1371/journal.pone.0112945)
Supplement: Figure S1 — Schematic diagram of the alterations in KP metabolism in cultured human primary glioma. Schematic diagram of the KP in cultured human primary glioma in the presence of IFN-γ. Arrows indicate significant metabolite over/under - production in response to IFN-γ stimulation for 48 and 72 hours compared to untreated. Arrows indicate significant enzyme up/down-regulation in response to IFN-γ stimulation for 24 hours compared to untreated. IFN-γ stimulation significantly potentiated the expression of IDO-1 IDO-2, KYNU, KMO and significantly down-regulated ACMSD and KAT-I expression. This significantly increased KP activity but significantly lowered the KYNA/KYN neuroprotective ratio. (DOCX) [file pone.0112945.s001.docx]

**SUPPORTING INFORMATION**


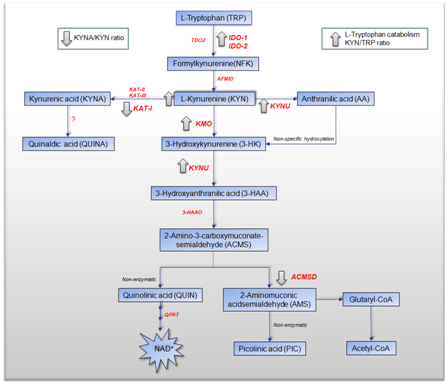


Figure S1: Schematic diagram of the alterations in KP metabolism in cultured human primary glioma. Schematic diagram of the KP in cultured human primary glioma in the presence of IFN-γ. Arrows indicate significant metabolite over/under - production in response to IFN-γ stimulation for 48 and 72 hours compared to untreated. Arrows indicate significant enzyme up/down-regulation in response to IFN-γ stimulation for 24 hours compared to untreated. IFN-γ stimulation significantly potentiated the expression of IDO-1 IDO-2, KYNU, KMO and significantly down-regulated ACMSD and KAT-I expression. This significantly increased KP activity but significantly lowered the KYNA/KYN neuroprotective ratio.
