# Supplementary material for: Involvement of the Kynurenine Pathway in Human Glioma Pathophysiology
Source: PLoS One. 2014 Nov 21;9(11):e112945. doi: 10.1371/journal.pone.0112945 (PMC4240539; doi:10.1371/journal.pone.0112945)
Supplement: Table S1 — qRT- PCR forward and reverse primer sequences and other parameters. (DOCX) [file pone.0112945.s002.docx]

**SUPPORTING INFORMATION**

**Table S1:** qRT- PCR forward and reverse primer sequences and other parameters

| Primer name | Accession number | Sequence  Forward primer 5’-3’ | Sequence  Reverse primer 5’-3’ | Amplicon size (bp) | Annealing Temperature (^o^C) | Source | | | Efficiency (%) |
| --- | --- | --- | --- | --- | --- | --- | --- | --- | --- |
| HPRT | NM_000194.2 | TGAGGATTTGGAAAGGGTGT | GAGCACACAGAGGGCTACAA | - | 60 | | [[57](#_ENREF_57)] | 97.8 | |
| IDO-1 | NM_002164.4 | tcatctcacagaccacaagtca | gcaagaccttacggacatctcc | 107 | 60 | | PrimerBank ID 4504577a2 | 99 | |
| IDO-2 | - | aagatagaggatgctgacaata | tccgttcccatatcattaact | - | 60 | | Dr. Helen Ball,  University of  Sydney | 115 | |
| TDO2 | NM_005651.2 | ATTCATAAGGATTCAGGCTAAAG | TTTCTCATCAAATAAGGACAGTAG | - | 60 | | - | 97 | |
| AFMID |  | CAAGTCAATGCTCAGAGATG | CCTGGGTGACAGAGTAAG | - | 60 | | - | 96 | |
| KAT-I | NM_001122671.1  NM_004059.4 | CACCACTGACGAAGATCCTGG | CTGAGCGGGTCTATCTCCTGA | 67 | 60 | | PrimerBank ID 4757928a1 | 110 | |
| KAT-II | NM_182662.1 | ggctggtggcttaccaaatc | actcggagaatactgaagtgctc | 124 | 65 | | PrimerBank ID 33469970a2 | 102 | |
| KAT-III | NM_001008661.1  NM_001008662.1 | cgctgatgtgtctttgctagatcc | cagaatgctgaaacggggatgg | 88 | 60 | | Integrated Sciences Dr. Magnino | 102 | |
| KYNU | NM_001032998.1  NM_003937.2 (Fwd only) | tgttcagtggggtgcattttt | aactccgatctcgcaggttta | 259 | 60 | | PrimerBank ID 12654129a3 | 106 | |
| 3-HAAO | - | ACATCGAAGAGGGTGAAG | GTGTTGGCAAACCTCTGT | - | 60 | |  | 90 | |
| KMO | NM_003679.3 | gcatctactaggtgacagccactg | aactctgccaggaagagccttatc | - | 65 | | Integrated Sciences Dr. Magnino | 106 | |
| ACMSD | - | CAGCACCGTTGTGAGCTAC | TGCCGCATAGACAGGAAAGAG | 187 | 60 | | [[58](#_ENREF_58)] | 103.3 | |
| QPRT | - | TTCACCTCTGCTCATCTC | CCTCACTATGTGCTCATTATC | - | 60 | | - | 96 | |
