# Supplementary material for: Involvement of the Kynurenine Pathway in Human Glioma Pathophysiology
Source: PLoS One. 2014 Nov 21;9(11):e112945. doi: 10.1371/journal.pone.0112945 (PMC4240539; doi:10.1371/journal.pone.0112945)
Supplement: Table S2 — Summary of primary and secondary antibody parameters used in ICC analysis. (DOCX) [file pone.0112945.s003.docx]

**SUPPORTING INFORMATION**

**Table S2:** Summary of primary and secondary antibody parameters used in ICC analysis

| TYPE | ANTIBODY | COMPANY | ISOTYPE | DILUTION |
| --- | --- | --- | --- | --- |
| Astrocytic marker | GFAP | Sigma | Polyclonal IgG | 1:200 |
| Astrocytic marker | GFAP | DAKO | Polyclonal IgG | 1:200 |
| Astrocytic marker | GFAP | Novocastra | Monoclonal IgG | 1:200 |
| Microglial marker | CD68 [KP1] | Abcam | Monoclonal IgG | 1:100 |
| Microglial marker | CD11b | Novus | Polyclonal IgG | 1:100 |
| Alexa Fluor 488 (green) | Secondary antibody | Invitrogen | Goat anti-rabbit IgG | 1:200 |
| Alexa Fluor 594 (red) | Secondary antibody | Invitrogen | Goat anti-mouse IgG | 1:200 |
| Alexa Fluor 488 (green) | Secondary antibody | Invitrogen | Goat anti-mouse IgG | 1:200 |
